# A Critical Review of Mechanical Ventilation Virtual Simulators: Is It Time to Use Them?

**DOI:** 10.2196/mededu.5350

**Published:** 2016-06-14

**Authors:** Juliana Arcanjo Lino, Gabriela Carvalho Gomes, Nancy Delma Silva Vega Canjura Sousa, Andrea K Carvalho, Marcelo Emanoel Bezerra Diniz, Antonio Brazil Viana Junior, Marcelo Alcantara Holanda

**Affiliations:** ^1^ Federal University of Ceara Medicine Federal University of Ceara Fortaleza Brazil

**Keywords:** positive-pressure respiration, medical education, computer simulation, learning

## Abstract

**Background:**

Teaching mechanical ventilation at the bedside with real patients is difficult with many logistic limitations. Mechanical ventilators virtual simulators (MVVS) may have the potential to facilitate mechanical ventilation (MV) training by allowing Web-based virtual simulation.

**Objective:**

We aimed to identify and describe the current available MVVS, to compare the usability of their interfaces as a teaching tool and to review the literature on validation studies.

**Methods:**

We performed a comparative evaluation of the MVVS, based on a literature/Web review followed by usability tests according to heuristic principles evaluation of their interfaces as performed by professional experts on MV.

**Results:**

Eight MVVS were identified. They showed marked heterogeneity, mainly regarding virtual patient's anthropomorphic parameters, pulmonary gas exchange, respiratory mechanics and muscle effort configurations, ventilator terminology, basic ventilatory modes, settings alarms, monitoring parameters, and design. The Hamilton G5 and the Xlung covered a broader number of parameters, tools, and have easier Web-based access. Except for the Xlung, none of the simulators displayed monitoring of arterial blood gases and alternatives to load and save the simulation. The Xlung obtained the greater scores on heuristic principles assessments and the greater score of easiness of use, being the preferred MVVS for teaching purposes. No strong scientific evidence on the use and validation of the current MVVS was found.

**Conclusions:**

There are only a few MVVS currently available. Among them, the Xlung showed a better usability interface. Validation tests and development of new or improvement of the current MVVS are needed.

## Introduction

### Mechanical Ventilation

Mechanical ventilation (MV) is a life support intervention used for patients in acute and/or chronic respiratory failure. The proper use of MV can decrease mortality in many diseases, such as acute respiratory distress syndrome (ARDS), chronic obstructive pulmonary disease, and others. Furthermore, good practices in MV can also reduce the length of intensive care unit (ICU) stay and decrease complications and hospital costs [1-5]. Over the past 20 years, rapid technological developments have led to significant improvements in MV, with the emergence of new microprocessor equipment, ventilatory modes, advanced features, and complex human-machine interfaces. However, this has been accompanied by underutilization of the available tools and difficulties in teaching about the equipment functioning and the handling of these devices by students and health professionals. Errors in mechanical ventilatory support may cause serious adverse events, even death [6,7]. A few studies have shown that medical residents, despite being responsible for the management and care of patients on ventilatory support, have difficulties in applying current knowledge in practical situations. Insufficient training on MV is a possible reason for this problem [5-8]. Teaching MV at the bedside with real patients is always a challenge. There are logistical problems, such as limited space in the ICUs, limited number of patients and clinical scenarios, and intrinsic risks related to the environment of the ICU. Furthermore, related examinations, such as arterial blood gases (ABG) analysis, are not always available at the point of care [7]. Given this context, it is believed that new teaching approaches are needed to contribute to better training in the use of MV.

### Simulation as a Powerful Teaching Tool for Health Professionals

Simulation techniques have been increasingly used as a learning method with advantages over the traditional ones [9,10]. Medical simulation can be defined as the use of a device for simulating a real-life situation in a patient for the purpose of education and research [11,12]. Two systematic reviews [13,14] have shown that medical simulation is effective for the acquisition of skills and to encourage better care of patients. Realistic simulations use real ventilators, usually connected to mannequins or mechanical simulators instead of patients. Although this approach reduces the risk for patients and may expand the number of mechanical clinical situations that can be taught, it still has logistic limitations, as the ventilators and the simulators are usually expensive and not easily accessible [9]. Easy access to the Web fostered the emergence of teaching tools as computer-assisted learning. This type of technological resource can improve the learning of medical students, particularly when related to the virtual simulation in health care. In fact, with the current technology, it may be possible to develop and provide Web-based access to virtual MV training to a massive number of students, teachers, and health care professionals worldwide at a relatively low cost [15,16]. Considering the limitations of both, MV training in ICU and even, realistic simulations, a good complementary approach would be to use virtual Web-based simulation of MV.

Therefore, mechanical ventilation virtual simulators (MVVS) arise as potential teaching tools that could help the implementation of ubiquitous learning on MV [17,18,19]. Their main advantages would be to promote student training in complex clinical scenarios, to predict and anticipate failures in procedures, to reduce the cost involved in acquisition and maintenance of medical equipment and materials, and to optimize training time and opportunity for continuous Web-based staff training. In addition, it presents zero risk for patients and provides more efficient and safer learning environments, familiarizing the students with the handling of the devices [9,17,18,20]. Despite that, use of MVVS is not the standard practice in MV training nowadays. Considering the complexity of MV practice and teaching and the fact the virtual simulation is only in its dawn, the hypothesis of this study were (1) there are only a few available Web-based MVVS; (2) they are not yet validated for medical training; and (3) furthermore, their usability as a teaching tool is unknown and may differ substantially. We aimed to identify and describe in detail the current available MVVS, to compare their features regarding their functionalities on usability tests related to their viability as teaching tools and to review the literature on validation studies.

## Methods

This is a descriptive, quantitative, and exploratory study, aimed to identify articles that investigated MVVS as a teaching tool. A comparative evaluation of the simulators was performed, based on a literature/Web review followed by usability tests by *experts* users [21,22].

### Question

We sought to answer the following questions: What are the current Web-based MVVS available? Are they ready for use in training MV? Are there validation studies on their usefulness for MV training?

### Systematic Review

#### Search Strategy

Electronic searches were performed in the Cochrane Library, PubMed, and Scielo databases, from April 1990 to April 2015. The search included the terms “computer simulation,” “simulator,” “medical education,” “learning,” “mechanical ventilation,” and the operator “AND” was used in all databases. This review included papers published in 3 languages (Portuguese, English, and Spanish).

#### Study Selection and Eligibility Criteria

We defined MVVS as the use of a device for simulating a real-life situation in a patient for the purpose of education and research [11,12]. Randomized and controlled clinical assays, prospective studies, and systematic review were preferred to investigate MVVS as a teaching tool.

To determine study eligibility, 3 investigators (GCG, JAL, and NDS) reviewed each article, the study title, and abstract, independently, and thereafter, the full text of the manuscript. The discrepancies were resolved by discussion among the review authors (MAH, AC, and ABV).

### Usability Tests

#### Convenience Sample

The sample was intentional, judgmental, and nonprobable, based on the assumption that the knowledge of the researcher on the population and its elements can be used to select the individuals to constitute the sample. Thus, 6 *experts* were purposively chosen from 2 categories: 3 physicians and 3 physiotherapists. We defined as *experts,* professionals who were university teachers or preceptors, also board-certified specialists in the professional category or who were coursing postgraduate program in critical care/pulmonology, and who have worked daily with mechanically ventilated patients for at least 5 consecutive years.

#### Study Design

Usability measures the efficacy, the efficiency, and the satisfaction with which the user can perform a specific set of tasks in a particular environment, mainly aiming to evaluate whether simple and basic tasks can be easily performed by the users [23].

First, each expert assessed the simulators by performing tasks, simulating 11 scenarios for invasive MVVS (Multimedia Appendix 1) and 6 scenarios for one noninvasive MVVS (Multimedia Appendix 2).

The second step aimed to assess the usability of each simulator by checking how well 10 heuristic principles [24] were met by the MVVS interface (Multimedia Appendix 3), through the application of a usability test. The experts assessed whether the MVVS meets the heuristics principles by the following scores according to a Likert scale: TD, totally disagree (1 point); D, disagree (2); N, neutral (3); A, agree (4); and TA, totally agree (5). The usability scores were obtained by computing the sum of the scores obtained for each heuristic principle analysis according to the evaluation of 6 experts. The maximum score to be obtained by a specific MVVS for one heuristic principle would be 5 points × 6 experts=30 points, and the minimum, 1 × 6=6 points. The total maximum score to be obtained by summing all scores for the 10 heuristic principles would be 10 heuristic principles × 30 points or each one=300 points and the minimum would be 10 × 6=60 points.

The third step consisted in the application of the Visual Analog Scale (VAS) to evaluate the easiness to use each MVVS. The VAS ranged from 0 (zero, very difficult to use) to 10 (ten, very easy to use) (Multimedia Appendix 4) [22]. Finally, the participants answered the following question: “Which simulator among those you tested would you recommend for teaching? Why?”

#### Time and Place

This study was performed in the Respiratory Laboratory, in the Biomedicine Center, Internal Medicine Department, Federal University of Ceara, Fortaleza, Ceara, Brazil. All experts assessed the MVVS in the same day, 3 in the morning and 3 in the afternoon. A Sony Vaio notebook with Windows 8 with wireless connection was used for high-speed Internet. During the tests, there were no problems with the Internet connection with no interruptions in the procedures. In the laboratory, the expert was seated in a comfortable chair, and they were acclimatized to the room temperature (22-23^°^ C, special attention to avoid noise or distractions was given. The sequence for testing the MVVS was randomized for each expert. A total time of 2 hours was given for each expert. Considering that only 6 MVVS were tested, a mean time of 20 minutes per simulator was used.

#### Ethical Precepts

The present study followed the ethical precepts established by the Resolution 466, 2012 by the National Health Council [25], fulfilling the requirements of the Free and Clarified Consent Term; that is, ensuring the rights of the subjects and allowing them to drop out of the study at any time. Therefore, in observance of the ethical principles, the participants were identified as E1, E2, E3, E4, E5, and E6.

### Statistical Method

The primary quantitative outcome was the score obtained for the 10 heuristic principles according to the answer of the experts to the usability questionnaire for the MVVS in executing specific predefined tasks. For analysis of each heuristic principle, the Kruskal–Wallis test was used. When a statistically significance was present a post hoc Mann–Whitney test was performed to compare pairs of MVVS, adjusting the significance level by the Bonferroni correction for multiple comparisons. For better visualization of the results concerning the total sum of scores for all heuristic principles, the results were expressed in percentage of the maximum of 300 points. A second quantitative outcome was the mean value for the score obtained in the VAS assessment. The significance level considered was *P*<.05 for a confidence interval of 95%. The statistical software SPSS 22.0 was used for all comparisons.

## Results

### Systematic Review

Eight MVVS accessible on the Web were found after extensive literature/web review: Beta (University of Pittsburgh, USA), Evita Trainer XL (Drager, Lubeck, Germany), Hamilton G5 (Hamilton Medical AG, Rhazuns, Switzerland), Inter Plus VAPS/GMX (Intermed Hospital Medical Equipment Ltda, Sao Paulo, Brazil), Servo 900C, Besim (Dr. Frank Fischer, Germany), Simulation-Based Educational Tool for Noninvasive Ventilation (NIV) (European Respiratory Society), Virtual Ventilator (Sagamihara/ Kanagawa, Kitasato University, Japan), and Xlung (Xlung, Fortaleza, Brazil).

The Simulation-Based Educational Tool for NIV was the only noninvasive MV simulator included in this study. This ventilation simulator integrates a NIV Competency Course of the European Respiratory Society. All the other MVVS were related to invasive MV.

**Figure 1:**
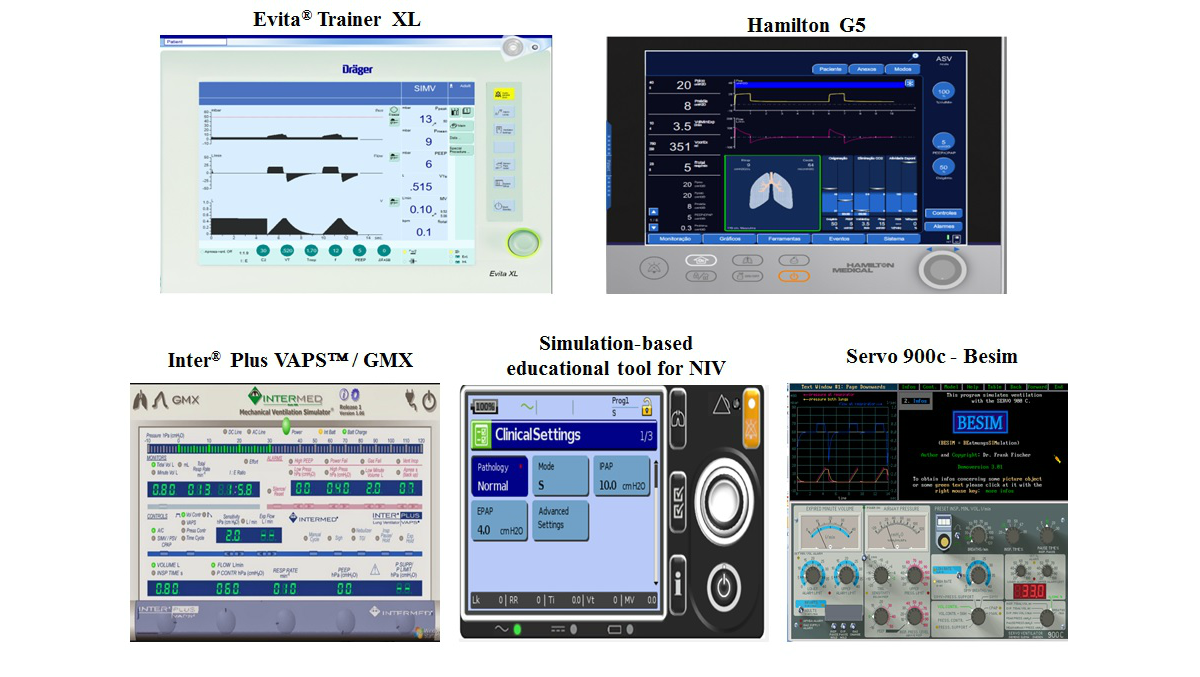
Screen shots of brand-type MVVS interfaces.

**Figure 2:**
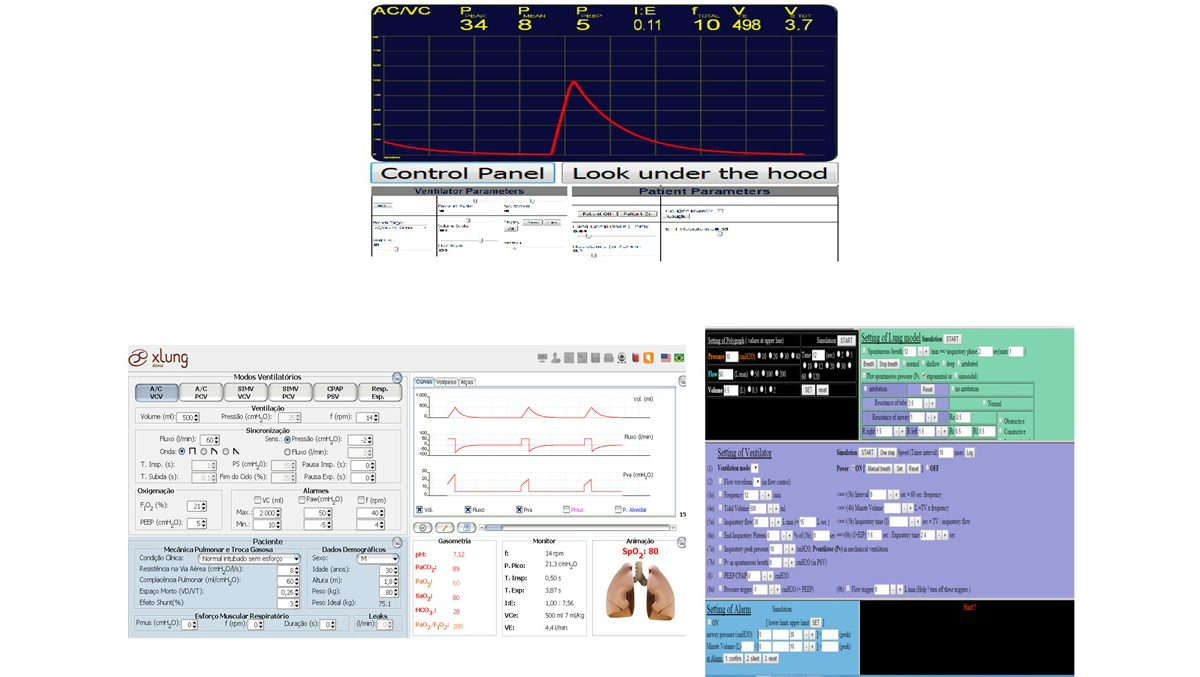
Screen shots of generic MVVS interfaces.

The MVVS were categorized as brand type or generic (not related to a particular brand), and their interfaces are shown in Figures 1 and 2. Brand-type MVVS (Servo 900C, Besim Evita Trainer XL, Hamilton G5, Inter Plus VAPS/GMX and Simulation-Based Educational Tools for NIV) are those that reproduce a mechanical ventilator interface of a particular company or brand, for training their staff, and disseminating knowledge of their equipment. Generic MVVS (Beta, Virtual Ventilator, and Xlung) are those developed as a teaching tool for improvement of user skills, in the field of MV in general. These simulators can be accessed for free downloads, Web-based use, or paid subscriptions.

**Figure 3:**
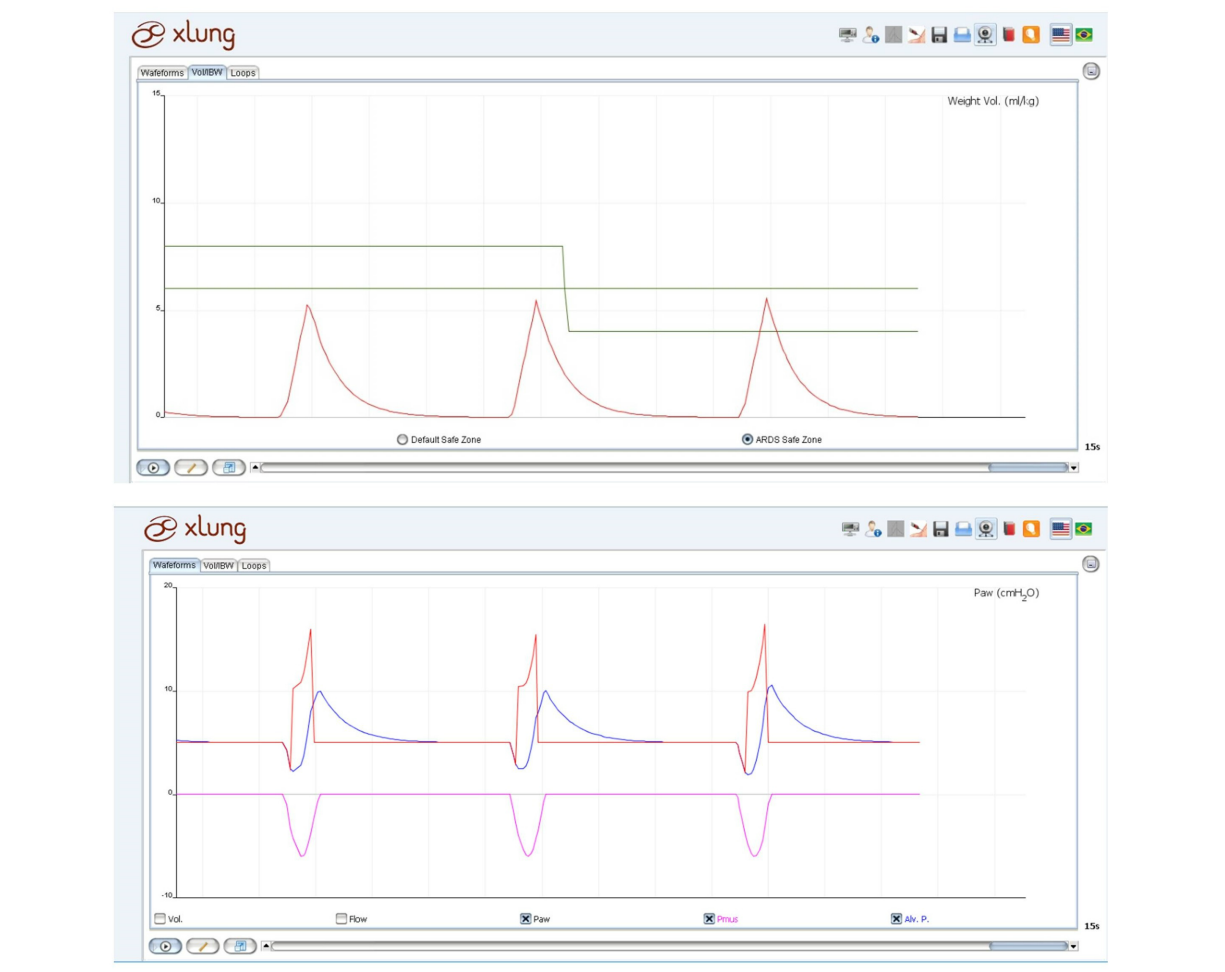
Special graphics of the Xlung MVVS. At the top, ponderal tidal volume according to the ideal body weight (mL/kg) with depicted safe zones and at the bottom, exhibition of airway (red), muscular (pink), and alveolar (blue) pressures altogether.

Windows (Microsoft Corporation) is the operating system compatible with all the MVVS mentioned previously. The Hamilton G5 simulator, the Simulation-Based Educational Tool for NIV, and the Xlung can also be used in the Mac OS X (Apple Inc.) system. The main characteristics of the MVVS are shown in the Multimedia Appendix 5. All the MVVS provide graphical monitoring of the airway pressure, flow, and volume × time curves. Although most of MVVS provide the F_I_O_2_ setting, only Xlung allows monitoring of SpO_2_ and ABGs of the “patient,” enabling the observation of the immediate effects of F_I_O_2_ changes on pulmonary gas exchange in real time. Monitoring capnography is found only in the Evita Trainer XL. Xlung has the option to display the respiratory muscular pressure and the alveolar pressure, which can be seen optionally in the pressure × time curve. It also shows the ponderal volume (tidal volume per kg of IBW), which is presented graphically with 2 safe zones, for ARDS or non-ARDS patients, that are shown in Figure 3. Regarding the configuration tools, the Hamilton G5 and the Simulation-Based Educational Tool for NIV offer 25 and 19 choices of languages, respectively. The Xlung allows the user to load and save the simulations already carried out to be accessed at a later time.

### Usability Tests

Six health professionals participated as *expert* users (3 physicians and 3 physiotherapists). Of the 8 simulators, 6 were evaluated. The Servo 900C, Besim and Virtual Ventilator were excluded from this part of the study. The former would only provide the DEMO version, not allowing the user to perform the necessary tasks; the latter was unavailable at the website http://info.ahs.kitasato-u.ac.jp/tkweb/tklsim2/indexE.html by the time of the usability test (April 15, 2016). Table 1 shows the tasks asked by the experts for usability evaluation of the MVVS performance.

**Figure 4:**
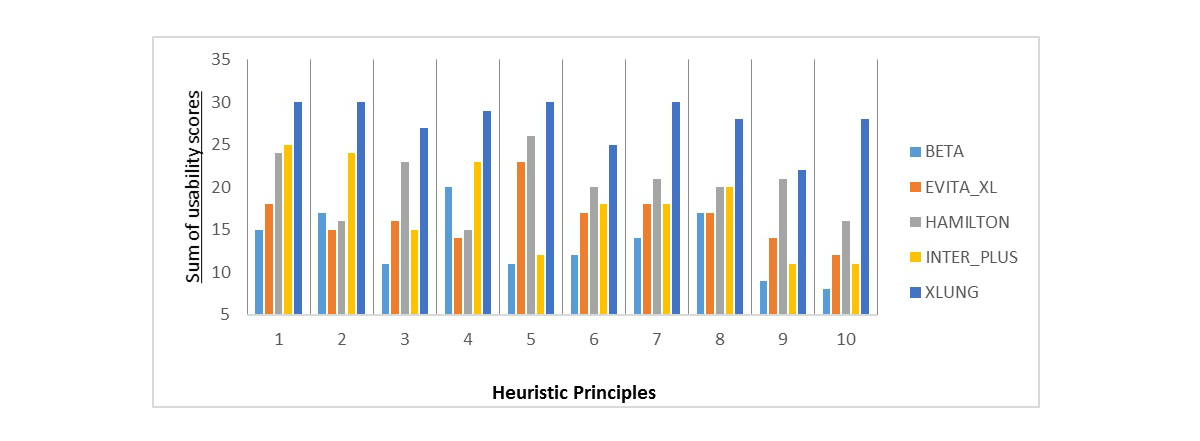
Scores obtained by the MVVS for each one of the heuristic principles. * *P*<.05 vs Xlung; + *P*<.05 vs Hamilton G5; 1: visibility of the system status; 2: correspondence between the system and the real world; 3: freedom and control by the user; 4: consistency and standards; 5: error prevention; 6: recognition instead of recall; 7: flexibility and efficiency in the utilization; 8: layout and minimalist design; 9: help the user to recognize, diagnose, and recover errors; and 10: help and documentation.

**Figure 5:**
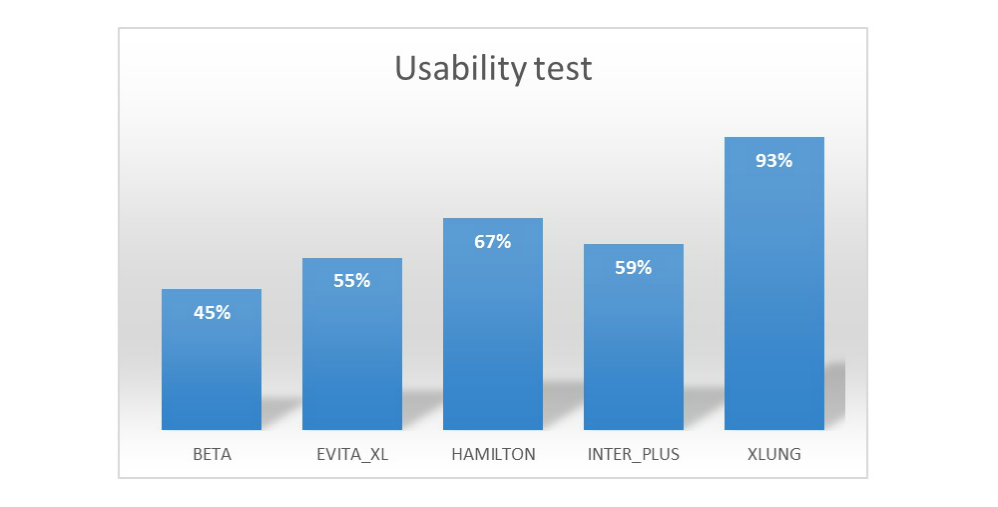
Performance of 5 MVVS as a percentage of the maximum obtainable scores (300 points) of their interfaces usability for the assessment of the 10 heuristic principles altogether.

Figure 4 shows the scores obtained by the MVVS for each one of the heuristic principles of their interfaces according to the *experts* assessments. Figure 5 shows the performance of 5 MVVS as a percentage of the maximum obtainable scores (300 points) for the assessment of the 10 heuristic principles altogether. The mean VAS scores for the easiness of use for the MVVS were the following: Xlung=9, Hamilton G5=7, Inter Plus VAPS/GMX and Evita Trainer XL=6, and Beta=4. When asked about the question: “Which simulator among those you tested would you recommend for teaching? Why?” all the experts chose Xlung, with the following answers:

E1: *“It is an easy to manipulate simulator, simulation is realistic and predicts iatrogenic MV complications”*; E2: *“Easy to use, didactic and intuitive design, complete capability of interaction giving the student innumerous possibilities of simulation with feedback by the results obtained on the arterial blood gases”*; E3: *“Easy to access and manipulate, simple and intuitive language with various possibilities of adjustments and includes arterial blood gases and pulse oxymetry, enabling the user a more reliable simulation of a real case scenario”*; E4: *“Simple and complete interface with various possibilities of clinical scenarios and available parameters”*; E5: *“A great teaching tool on MV, good interaction between student and teacher, in different clinical situations”*; and E6: *“It features various parameters and tools, simple language, with important possibilities of adjustments, such as monitoring of gas exchange and respiratory mechanics.”*

**Table 1:** Usability tasks as evaluated by experts in each simulator.

Tasks	Beta	Evita Trainer XL	Hamilton G5	Inter Plus	Xlung
English language	✓	✓	✓	✓	✓
TV^b^/weight adjustment	✓	✓	✓	✓	✓
AC/VCV^c^ adjustment	✓	✓	✓	✓	✓
Calculate plateau	✓	✓	✓	✓	✓
Identify auto-PEEP^d^	X	✓	✓	✓	✓
AC/PCV^e^ adjustment	✓	X	✓	✓	✓
F_I_O_2_^f^ adjustment	✓	✓	✓	X	✓
Curves (vol, flow, paw)	✓	✓	✓	✓	✓
Max pressure alarm	X	✓	✓	✓	✓
PSV^g^ adjustment	✓	✓	✓	✓	✓
Save simulation	X	X	X	X	✓

^a^✓ denotes accessed by the experts; X denotes not found or accessed by the experts.

^b^TV, tidal volume.

^c^AC/VCV, assist/control with volume cycling.

^d^PEEP, positive end expiratory pressure.

^e^AC/PCV, assist/control with constant pressure, timed cycling.

^f^F_I_O_2_, fraction of inspired oxygen.

^g^PSV, pressure support ventilation.

Simulation-Based Educational Tool for NIV was also evaluated by *experts*, but was not included in the statistical tests, because there was no other NIV MVVS for comparison. Regarding the tasks, does not allow adjustment of F_I_O_2_ and does not offer the option to save the simulation. Presented 65% (195 points) of its interface usability for the assessment of the 10 heuristic principles altogether of the maximum obtainable scores (300 points) and obtained mean score 6.9, according to the VAS.

After an extensive search for papers in the literature, no scientific evidence on the use and validation of the current MVVS was found. Only Xlung was tested to evaluate the teaching of MV principles to fourth-year medical students. In that study, 2 educational activities were analyzed. The first one evaluated 23 undergraduate medical students on the usability of the Xlung in a computer laboratory. In the second, 24 other students had simulation-based activities with the software. After the first activity, 75% of the students agreed with the statement: “I learned from the simulation aspects not previously understood in theory and practice”; and 78% of them agreed that: “The simulator creates a better understanding of how to adjust the ventilator.” In the second activity, there was a statistically significant increase in correct answers in a standardized test [26].

## Discussion

### Principal Findings

The literature/web review of the currently available Web-based MVVS identified 8 simulators. They showed diversity, mainly regarding the nomenclature of the ventilatory modes, adjustments of the ventilatory settings, and the monitoring of pulmonary gas exchange parameters. The Hamilton G5 and the Xlung covered a broader number of parameters, tools, and have easier Web-based access. Except for the Xlung, none of the simulators displayed monitoring of ABGs and alternatives to load and save the simulation. The Xlung performed all the usability tasks, obtained the greater scores on heuristic principles and the greater mean score on the VAS, and it was the preferred one for teaching purposes.

To our knowledge, this is the first study that assessed the usability of the interface of Web-based MVVS. It is important to mention that it was not aimed to perform a market analysis. In fact, this type of study addresses the attractiveness and the dynamics of a particular brand within a particular industry [27]. In this investigation, the simulators were classified into brand type and generic. This classification aimed to separate the simulators that could reproduce the mechanical ventilator interface of a particular brand from those developed as a teaching tool for improvement of user skills.

For efficient use of MVVS as a teaching tool, it is essential to set up the typical parameters of the respiratory physiology according to clinical scenarios or diseases that are observed in real life. Except for Evita Trainer XL, the simulators offer options of different clinical scenarios. The great advantage of this is the possibility of modeling patient characteristics, which may assist in the teaching and learning of MV in certain pathologies. The Evita Trainer XL, Hamilton G5, and Xlung allow determination of the IBW. There is no doubt about the importance of calculating the IBW according to the height and gender of the patient. Actually, the utilization of this parameter for promoting safe and effective ventilation is essential. Only Xlung allows monitoring of SpO_2_ and ABGs of the “patient,” enabling the observation of the effects of F_I_O_2_ changes on pulmonary gas exchange in real time. This interesting feature makes it possible to calculate the PaO_2_/F_I_O_2_ ratio, which is important to teach how to quantify the severity of lung injury [19]. There is much heterogeneity of the functional characteristics and usability among the MVVS. The test of interface usability displays some methods for analysis: “formal,” “automatic,” “empiric,” and “heuristic” (or “analytic”). The heuristic evaluation is a method for inspection, where an *expert* interacts with the interface and assesses it according to usability principles previously defined, the so-called heuristic principles [24,28,29].

The heuristic evaluation is considered the best inspection method to predict problems, usually serious, faced by the users [30]. It has been also used on *sites,* teaching resources, and *software* [31-38]. When used in mechanical ventilators, it allows the identification of usability problems [21]. In this study, we tested individually each MVVS, regarding the usability tasks, the heuristic principles questionnaire, and the VAS scores. It was noted that all of them still have limitations. There are problems in relation to the absence of important parameters, confusing terminology of the ventilatory modes, and the usability of some functions. In some of them, the layout of the screen frequently has too much information and the location and function of the “buttons” are not always intuitive. For example, the patient ventilation settings, curves, and monitoring data are not always separated for a better view.

The layout of the traditional ventilatory modalities is easier to be handled by the user. Under the classificatory point of view, a consensus or an international standardization is necessary, as a mode can have different names in different ventilators. These new ventilatory modalities emerge for the association of other basic modalities; however, they can be little or poorly used, according to the experience of the professional. Over the last years, some authors have tried to standardize the system for the classification and description of the ventilatory modalities [39-41].

Monitoring the respiratory mechanics was another critical item assessed in the MVVS, for being useful both for the diagnosis of the subjacent condition of the patient and for an individualized adjustment. There is marked heterogeneity among the MVVS. The Beta, Hamilton G5, and Servo 900C, Besim only displayed the value of the plateau pressure during monitoring, not allowing the user to calculate it. However, the other simulators allowed for the adjustment of the inspiratory pause. This parameter is fundamental for the evaluation of the respiratory mechanics, and it is necessary for the measurement of the plateau pressure, which is used in the calculation of compliance, airway resistance, and driving pressure [19].

The real-time cycle-to-cycle monitoring of the flow, volume, and pressure curves, in addition to pressure-volume and volume-flow loops, allows the collection of qualitative data and the accurate calculation of the airflow resistance and compliance of the respiratory system. A differential in the simulators Evita Trainer XL, Hamilton G5, and Xlung is the option pressure × volume and flow × volume loops. Its qualitative analysis allows the diagnosis of problems on respiratory mechanics [42,43].

This study has limitations. It is impossible to be completely sure about not including other existing virtual simulator in this study. However, we believe to have included the most important MVVS considering our thorough search methodology. Great effort was made to reduce the bias of favoring Xlung as we acknowledge that there are conflicts of interest regarding the authors. However, in an attempt to minimize these conflicts, we performed a usability test of all simulators by independent *experts* on MV. Considering that MV is a very complex type of support, we also recognize that only a limited number of relatively simple tasks and scenarios were tested for usability assessment and the number of experts was small. Even so, important and significant differences among the MVVS were detected.

The present work has practical important implications. We now emphasize that, as a tool to MV teaching, the ideal MVVS should offer the following features: facilitate and stimulate the comprehension of the MV handling through the following characteristics: easiness to access, good usability, capability of reproducing the most common scenarios found in practice, have a friendly interface, and allow the user to create, save, and share simulations. To develop a good MVVS, it is necessary to elaborate a design focusing the different types of users, both teachers and students. Training MV with MVVS is essential for students, as it provides them with the opportunity to practice in a safe environment, where MV can be handled reproducing the real life scenarios, with no risks for the user or, even more importantly, for the patients.

Although promising, versatile, and far-reaching it is important to recognize that the use of MVVS as both a computer-assisted learning and a simulation technique, it may still have high costs because the need for technical support from software programmers and critical care specialists consultation and training of faculties. Expand this kind of learning requires cultural changes, planning, financing, multidisciplinary work, and effective quality control [15,44].

### Conclusion

In conclusion, there are only a few MVVS currently available. Among them, the Xlung showed a better usability interface. Validation tests and development of new or improvement of the current MVVS are needed.

## Appendix

Multimedia Appendix 1 Tasks assessed by the users while handling a MVVS, simulating invasive MV scenarios.

Multimedia Appendix 2 Tasks assessed by the users while handling a MVVS, simulating non-invasive MV scenarios.

Multimedia Appendix 3 Usability test with 10 heuristic principles.

Multimedia Appendix 4 Visual Analog Scale to evaluate easiness to use MVVS.

Multimedia Appendix 5 The main characteristics of the MVVS.

## Abbreviations

ABG: arterial blood gases
ARDS: acute respiratory distress syndrome
FIO2: fraction of inspired oxygen
IBW: ideal body weight
ICU: intensive care unit
MV: mechanical ventilation
MVVS: mechanical ventilation virtual simulators
NIV: noninvasive ventilation
VAS: Visual Analog Scale
SpO2: pulse oxygen saturation
