# Supplementary material for: A Critical Review of Mechanical Ventilation Virtual Simulators: Is It Time to Use Them?
Source: JMIR Med Educ. 2016 Jun 14;2(1):e8. doi: 10.2196/mededu.5350 (PMC5041346; doi:10.2196/mededu.5350)
Supplement: Multimedia Appendix 1 [file mededu_v2i1e8_app1.pdf]

Multimedia Appendix 1: Tasks assessed by the users while handling a MVVS, simulating invasive MV scenarios

1. Check whether English language is available
2. Adjust tidal volume for a male adult patient, 70kg
3. Considering the settings for the patient in task 2, adjust to VCV mode
4. Calculate the plateau
5. Calculate and identify the presence of auto-peep
6. Considering the settings for the patient in task 2, adjust to PCV mode
7. Adjust the F_IO_2 to 50% and verify the PaO_2 and SpO_2
8. Indicate the volume, pressure and flow curves
9. Set the maximum pressure alarm
10. Considering the settings for the patient in task 2, adjust to PSV mode
11. Save the simulation
