# Supplementary material for: A Critical Review of Mechanical Ventilation Virtual Simulators: Is It Time to Use Them?
Source: JMIR Med Educ. 2016 Jun 14;2(1):e8. doi: 10.2196/mededu.5350 (PMC5041346; doi:10.2196/mededu.5350)
Supplement: Multimedia Appendix 2 [file mededu_v2i1e8_app2.pdf]

Multimedia Appendix 2: Tasks assessed by the users while handling a MVVS, simulating non-invasive MV scenarios

1. Check whether English language is available
2. Adjust to CPAP mode
3. Adjust to S/T mode
4. Adjust the IPAP and EPAP
5. Adjust the F_IO_2 to 50% and verify the PaO_2 and SpO_2
6. Indicate the volume, pressure and flow curves
7. Set the maximum pressure alarm
8. Save the simulation
