# Supplementary material for: A Critical Review of Mechanical Ventilation Virtual Simulators: Is It Time to Use Them?
Source: JMIR Med Educ. 2016 Jun 14;2(1):e8. doi: 10.2196/mededu.5350 (PMC5041346; doi:10.2196/mededu.5350)
Supplement: Multimedia Appendix 3 [file mededu_v2i1e8_app3.pdf]

Multimedia Appendix 3: Usability test with 10 heuristic principles

	TD	D	N	A	TA
**1. Visibility of the System status**					
The system must provide the users with accurate and fast information about what is happening; i.e., appropriate *feedback*	1	2	3	4	5
**2. Correspondence between the system and the real world**					
The system must speak the users' language, using words, expressions and concepts that are familiar to them, following the real world conventions, and presenting the information in a natural and logical order	1	2	3	4	5
**3. Freedom and control by the user**					
An "emergency exit" is needed, clearly marked, in order to allow exiting from any undesired state without having to go through an extensive dialogue	1	2	3	4	5
**4. Consistency and standards**					
Words, situations or actions must always mean the same, without making the users uncertain about their meaning. The same action must never be identified by different icons or words	1	2	3	4	5
**5. Error prevention**					
Better than efficient error messages are an efficient and careful *design,* preventing problems and eliminating error-prone situations. A confirmation option is mandatory before executing any critical action	1	2	3	4	5
**6. Recognition instead of recall**					
Minimize the user's memory load, making visible the options to perform certain actions. Instructions on how to use the system must be visible or easily recoverable. The interface must offer contextual help and information to orient the user actions; i.e., the system must dialogue with the user	1	2	3	4	5
**7. Flexibility and efficiency in the utilization**					
The system must be easy to be used by lay users, but flexible enough to become agile to advanced users. This flexibility can be attained with permission to shortcut keys	1	2	3	4	5
**8. Layout and minimalist *design***					
Avoid the interface to say more than the user needs to know. The system must be simple, straight and natural	1	2	3	4	5
**9. Help the user to recognize, diagnose and recover errors**					
The error messages must be expressed in simple language (without codes), indicate the problem with precision and suggest a constructive solution	1	2	3	4	5
**10. Help and documentation**					
Even the best system must provide help and documentation options. This information must be easily found through a search tool	1	2	3	4	5

1- TD: totally disagree; 2- D: disagree; 3- N: neutral (a); 4- A: agree; 5- TA: totally agree
