# Supplementary figures and images for: A Critical Review of Mechanical Ventilation Virtual Simulators: Is It Time to Use Them?
Source: JMIR Med Educ. 2016 Jun 14;2(1):e8. doi: 10.2196/mededu.5350 (PMC5041346; doi:10.2196/mededu.5350)

Multimedia Appendix 4: Visual Analog Scale to evaluate easiness to use MVVS

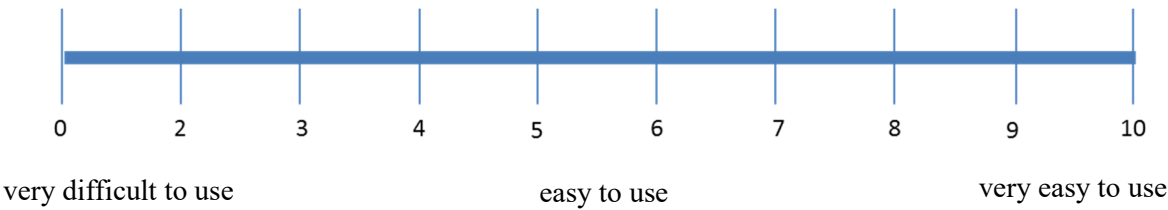

**SCORE:** \_\_\_\_\_

Supplement: Multimedia Appendix 4 [file mededu_v2i1e8_app4.pdf]
