# Supplementary material for: A Critical Review of Mechanical Ventilation Virtual Simulators: Is It Time to Use Them?
Source: JMIR Med Educ. 2016 Jun 14;2(1):e8. doi: 10.2196/mededu.5350 (PMC5041346; doi:10.2196/mededu.5350)
Supplement: Multimedia Appendix 5 [file mededu_v2i1e8_app5.pdf]

Multimedia Appendix 5: The main characteristics of the MVVS.

The Hamilton G5 and the Xlung™ cover more variables in terms of anthropomorphic patient data (gender, height, ideal body weight and age). Just 3 MVVS (Evita® Trainer XL, Hamilton G5 e Inter® Plus VAPS™/GMX) offer neonatal and pediatric simulation options besides adult patient. Evita® Trainer XL, Hamilton G5 and Xlung™ allow determination of the Ideal Body Weight (IBW). Except for the Servo 900C - Besim, the MVVS reproduce clinical scenarios of "normal", "restrictive" or "obstructive" types of respiratory mechanics of the patients. The Hamilton G5, Inter® Plus VAPS™/GMX, Virtual Ventilator and Xlung™ can configure the compliance and airway resistance of the virtual patient. The Inter® Plus VAPS™/GMX and Xlung™ may adjust intensity, duration and rate of the patient respiratory muscle effort. Only Xlung™ allows the configuration of the amount of shunt and Vd/Vt (Dead Space/Tidal Volume ratio) for pulmonary gas exchange abnormalities.

Most of the MVVS offer traditional ventilatory modes, such as Assist / Control with Volume Cycling (AC-VCV), Assist/Control with Constant Pressure, Timed Cycling (AC-PCV), Synchronized Intermittent Mandatory Ventilation (SIMV) with Pressure Support Ventilation (PSV), and Continuous Positive Airway Pressure (CPAP). The Virtual Ventilator simulator presents other ventilatory modes, such as the Pressure-Regulated Volume Control (PRVC), Intermittent Mandatory Ventilation (IMV) and Mandatory Minute Ventilation (MMV). In the simulator Evita® Trainer XL, the PSV mode, for example, is called CPAP/ASB (Assisted Spontaneous Breathing). The Hamilton G5 features the modes P/CMV, referring to the Pressure-Controlled Mandatory Ventilation, and S/CMV for the Synchronized Controlled Mandatory Ventilation. The Evita® Trainer XL also features Airway Pressure Release Ventilation (APRV), Proportional Pressure Support (PPS) and Intermittent Positive Pressure Ventilation (IPPV). The Hamilton G5 and Xlung™ have the option of spontaneous breathing, allowing simulation of monitoring patients without ventilatory support, as in the spontaneous breathing trial procedures.
